# Editorial: Rising stars in microbial physiology and metabolism: 2022

**DOI:** 10.3389/fmicb.2023.1254900

**Published:** 2023-07-18

**Authors:** Nicole R. Buan, Ulrike Kappler

**Affiliations:** ^1^Department of Biochemistry, University of Nebraska-Lincoln, Lincoln, NE, United States; ^2^School of Chemistry and Molecular Biosciences, The University of Queensland, Saint Lucia, QLD, Australia

**Keywords:** physiology, metabolism, microbe, microbiology, systems

This Research Topic was initiated to highlight work by young authors, the rising stars in the field of microbial physiology and metabolism. Microbial physiology and metabolism is an interdisciplinary field of research that seeks to uncover how the metabolic pathways of a cell work together to determine cell fate and function, whether that be growth, replication, pathogenicity, predation, respiration and fermentation, homeostasis or death. Ultimately, researchers like the ones featured here seek to integrate biological information and physicochemical parameters to try to find the underlying rules governing microbial function so that we can understand, predict and design microbes and microbial communities to improve society.

The study of microbial physiology and metabolism is also essential to understanding microbial function across scales, from sub-cellular information to the molecular level, to community and global ecological scales over time. Across all these levels, cells must conserve energy as individuals and as communities, doing so through competitive and cooperative relationships with each other, viruses, and their hosts. Microbial physiology and metabolism is at the heart of all these interactions, and can help to answer questions like: why are certain microbes pathogens while their close relatives are not? How can we “encourage” microbes to reliably produce food and fuels? And even, could microbes survive in outer space?

The authors featured in this Research Topic are initiating their independent careers and training the next generation of microbial physiology and metabolism researchers. In “*Phage-resistant Pseudomonas aeruginosa against a novel lytic phage JJ01 exhibits hypersensitivity to colistin and reduces biofilm production”*, Wannasrichan et al. isolated, characterized and sequenced a novel *Pseudomonas aeruginosa Pbunavirus* phage JJ01. They also characterized several JJ01 phage-resistant mutants of *P. aeruginosa*, which escaped phage killing by acquiring mutations that decreased biofilm formation and colony diameter, increased or decreased growth rate in liquid medium, but also increased susceptibility to antibiotics. These findings raise the possibility that phage JJ01 could be used to sensitize antibiotic-resistant *P. aeruginosa* through a combined therapeutic treatment approach.

Another way to promote antibiotic sensitivity is to supply a sensitizing agent. In “*Alanine-mediated P cycle boosting enhances the killing efficiency of kasugamycin on antibiotic-resistant Xanthomonas oryzae”*, Guan et al. studied global metabolomic changes associated with resistance of the rice pathogen *Xanthomonas oryzae* to kasugamycin. GC-MS profiling and enzyme assays revealed kasugamycin resistance was linked to a depression of the P cycle. The investigators confirmed that artificial repression of the P cycle by furfural increased kasugamycin resistance, while addition of alanine reversed the effect by increasing flux through the alanine-pyruvate portion of the P cycle.

Liu et al. observed an intriguing connection between the nitrogen cycle and heavy metal resistance in fungi. In “*A fungus (Trametes pubescens) resists cadmium toxicity by rewiring nitrogen metabolism and enhancing energy metabolism*” they describe how the white rot fungus *T. pubescens* can be cocultured with rice seedlings to protect the seedlings from cadmium toxicity (Liu et al.). Using transcriptomics and metabolomic data, they concluded that cadmium exposure causes *T. pubescens* to down-regulate production of non-essential amino acids and to increase flux through the TCA cycle, which increases energy metabolism. Their work hints at new ways microbes may be harnessed to promote plant crop productivity and nutrition in agricultural settings.

Microbial resistance to heavy metals is also a desired trait in industrial processes. In “*Comparative analysis reveals the modular functional structure of conjugative megaplasmid pTTS12 of Pseudomonas putida S12: A paradigm for transferable traits, plasmid stability, and inheritance?”*, Kusumawardhani et al. explored the potential for IncP-2 megaplasmids such as pTTS12 to transfer desirable traits between industrially useful microorganisms. They showed that pTTS12 transmission from *P. putida* S12 to *P. putida* KT2440 simultaneously transferred the ability to produce indole, resistance to heavy metals, and tolerance to the solvent toluene. Despite these benefits, pTTS12 was found to be unstable in KT2440 due to an unknown metabolic burden. Future work is needed to identify barriers to maintaining pTTS12 in other industrially useful *P. putida* strains.

The investigators and their teams featured in this Research Topic continue the tradition of linking theoretical, computational, and experimental evidence together to understand how microbes evolve and adapt to growing under a wide variety of conditions, and we look forward to seeing the contributions of these researchers in coming years.

## Author contributions

NB drafted the manuscript. UK and NB edited the manuscript. All authors contributed to the article and approved the submitted version.

